# How to improve recruitment, sustainability and scalability in physical activity programmes for adults aged 50 years and older: A qualitative study of key stakeholder perspectives

**DOI:** 10.1371/journal.pone.0240974

**Published:** 2020-10-29

**Authors:** Andrew O’Regan, Enrique García Bengoechea, Amanda M. Clifford, Monica Casey, Stephen Gallagher, Liam Glynn, Ciaran Doyle, Catherine Woods

**Affiliations:** 1 Graduate Entry Medical School, Health Research Institute, University of Limerick, Limerick, Ireland; 2 Physical Activity for Health Research Cluster, Health Research Institute, Department of Physical Education & Sport Sciences, University of Limerick, Limerick, Ireland; 3 School of Allied Health, Health Research Institute, University of Limerick, Limerick, Ireland; 4 Department of Psychology, Health Research Institute, University of Limerick, Limerick, Ireland; 5 Health Research Board Primary Care Clinical Trial Network, Galway, Ireland; Edinburgh Napier University, UNITED KINGDOM

## Abstract

**Introduction:**

Physical inactivity among adults aged 50 years and over is a worldwide health concern. The objectives of the study were to investigate the perspectives of those involved with existing physical activity programmes on optimising recruitment, sustainability and scalability of physical activity programmes for adults aged 50 years and over.

**Methods:**

The study was conducted in Ireland’s Midwest region, where community-based physical activity programmes are delivered to groups by state-funded Local Sports Partnerships. Programme attendees, physical activity professionals and community advocates were recruited. One-to-one interviews and focus groups were conducted in 2018, recorded, transcribed and analysed by an interdisciplinary team experienced in qualitative research. Over a series of meetings, a thematic approach was used to code and analyse the transcripts, categorising data into higher order codes, themes and overarching themes with the purpose of making meaning of the data. Twenty-nine people participated in four focus groups and 18 participated in one-to-one interviews.

**Findings:**

Data analysis produced three overarching themes. “Age appropriate” explains how communication and the environment should be adapted to the needs of adults aged 50 years and older. “Culture and connection” refer to the interplay of individual and social factors that influence participation, including individual fears and insecurities, group cohesion and added value beyond the physical gains in these programmes. “Roles and partnerships” outlines how key collaborations may be identified and managed and how local ownership is key to success and scalability.

**Conclusion:**

Successful recruitment, sustainability and scalability require an understanding that the target population has unique needs that must be catered for when planning interventions, communicating messages and choosing personnel. The findings of this study can inform the development of community-based programmes to increase physical activity in adults aged 50 years and older.

## Introduction

Physical inactivity continues to be a worldwide cause of morbidity and mortality despite public health strategies to combat it [1]. Research reports that adults aged fifty years and older are most susceptible to chronic illness and that physical activity (PA) can have an important role in the prevention and management of these illnesses [2]. Physical, mental [3] and social [4] benefits of physical activity have been reported but the vast majority of adults do not meet the minimum recommended physical activity guideline levels (PAGL) of 150 minutes of moderate intensity or 75 minutes of vigorous intensity physical activity per week [5]. Studies indicate that, in the USA [2] and Britain [6], significant proportions of adults over 50 years are inactive and that inactivity increases with increasing age [7]. In Ireland, only 35% of adults aged 55–65 and 18% of adults over 75 years reach the minimum PAGL [8]. Certain groups (e.g. very old and lower socio-economic groups) are more at risk of inactivity [9] as well as being less likely to join or maintain involvement with programmes [10, 11]. In the context of ageing populations, physical inactivity is recognised as the most common modifiable risk factor for chronic illness [12] and recent research has reported that more physical activity at any level of intensity and less sedentary time are associated with less mortality for middle aged and older adults [13].

The World Health Organisation (WHO) recommend group interventions to improve physical activity levels for older adults [14] but evidence regarding their development and delivery remains inconclusive [4, 10, 15]. Recruitment of participants that are truly representative of the population for trials of physical activity interventions has been a challenge for research, with selection bias, whereby those that have higher incomes and have higher educational levels are more likely to enrol, limiting the reach of the research [16]. Researchers have recommended developing alternative strategies to recruit more vulnerable populations, including socially isolated older adults [17]. Expert reviews have also emphasised the importance of developing sustainability [18] and scalability [1] in order to bridge the gap between research and practice for public health interventions. In this context, scalability means that interventions should become embedded in the system so that their health benefits are maintained [1], and sustainability is defined as the “continued use of programme components and activities for the continued achievement of desirable outcomes” [19].  Intervention sustainability is an under-researched area [20] that should be considered at the early phases of a project [21]. Evidence to inform policy development should seek to understand the social context of the problem [22] but the experiences of participants are not usually incorporated into planning [23]. The WHO Global Action Plan on Physical Activity recommends engaging with “grassroots community” [24] and, in Ireland, national strategy for healthy ageing advises that the perspectives of people on policies that affect them as they age should be sought through more inclusive consultation [25].

In response to global and national reports of physical activity and ageing, the University of Limerick, in conjunction with health service and community collaborators, established the Move for Life study [26]. The aim of Move for Life is to increase physical activity levels among adults aged 50 years and older through a behavour change intervention and to test the intervention through a cluster randomised controlled trial. The Move for Life intervention is based on behaviour change, social support and group cohesion. Prior to intervention development, it was considered essential to conduct research exploring the perspectives of the groups involved in existing physical activity programmes. While published studies of physical activity involving adults exist [27], no qualitative study in this area combining perspectives of programme attendees, physical activity professionals involved in programme design and delivery and community advocates, has been reported.

The overall aim of this study was to inform the development of the Move for Life intervention to increase physical activity in adults aged 50 years and older, by investigating the experiences of programme attendees as well as those community advocates and professionals involved in design and delivery of existing programmes. Specific objectives were to investigate the key factors for recruitment, retention and scalability:

1. How can recruitment of adults aged 50 years and older be improved with specific emphasis on harder to reach groups (e.g. the very old, socially isolated and lower socio-economic groups)?
2. How can physical activity be sustained after the research has been completed?
3. How can physical activity programmes be upscaled so that they become embedded in public health strategies?

## Materials and methods

### Study design

A qualitative design was employed, utilising a combination of semi-structured one-to-one interviews and focus groups and using the criteria for reporting qualitative research (COREQ) as a guideline for all stages of the study (S1 Table) [28]. Ethical approval was granted by the University of Limerick Education and Health Sciences Research Ethics Committee (Reference no.: EHS_2018_01_04).

### Context

This formative research study formed the initial phase of the Move for Life study (MFL) [26]. MFL is a complex, community-based intervention that aims to develop a training programme for professional PA tutors, underpinned by principles of self-efficacy [29], self-determination theory [30] and group cohesion [31] to augment existing physical activity programmes to increase a PA outcome. In the context of limited resources, it was also hypothesised that a peer mentor, whereby a lay member of a PA group who is not a professional instructor, may assist in some way with the implementation of a programme.

Move for Life collaborates closely with Local Sports Partnerships (LSPs), whose programmes it augments. In Ireland, LSPs operate as state bodies tasked with delivering low cost physical activity programmes by professional instructors to the communities they serve. They operate throughout the country and are structured geographically, interfacing with other state organisations such as the health service and county councils as well as local community bodies, including active retirement groups and sports clubs. Programmes offered by the LSPs which are relevant to the participants of this study include: women on wheels, a 12-week supervised cycling programme for women; men on the move, a 12-week structured physical activity class for men; Go for Life, an eight week programme of interactive games for older men and women; and Get Ireland Walking, an eight week starter programme for sedentary men and women. The Go for Life programme also provides Physical Activity Leadership schemes that train older adults in the skills necessary to lead physical activity classes in their own localities. All of the aforementioned programmes are delivered by professional LSP instructors.

### Recruitment and participants

Study participants, comprised individuals from three distinct groups. The first, programme attendees, were identified after the MFL team mapped where attendees would be likely to come from and it consisted of adults who were attending other physical activity programmes or who had attended them in the past. The only exclusion criterion for this group was that participants aged under 50 years were not invited. The second group were physical activity professionals responsible for running programmes similar to MFL or similar though non-physical activity related programmes. The third stakeholder group were community advocates who were not directly involved in these programmes but, in either a voluntary or professional capacity, had prominent supportive roles in their communities and were involved in publicising or referring to PA interventions. It was agreed that if data saturation was reached at the data analysis stage no further recruitment would take place.

Recruitment was conducted by a combination of convenience sampling and snowballing. The LSPs had contact details for members of the programme attendee and physical activity professional groups and sent an invitation email to them. Programme attendees who were interested were asked to alert other members of their group who would not have access to email about the study. The MFL team mapped a list of community advocates and found their email addresses online and in local directories and the interviewer sent the invitation email to them. Those that were interested were invited to respond by email to the interviewer who subsequently sent them a detailed information sheet, and informed consent form that had space for them to outline their preferred logistical details for the interview, including timing and location. Only after written informed consent was given and a suitable time and location for the interview agreed did the interview take place. The study participants consisted of programme attendees who were adults aged 50 years and older who had attended community-based group PAPs facilitated by the Local Sports Partnerships, physical activity professionals who were involved in the design, development, administration or delivery of the programmes, and community advocates, involved in advocating for the rights of older adults to access quality services -not only physical activity–in their local community.

### Data collection

One-to -one interviews and focus groups were conducted by a member of the research team (MC) during January and February 2018. The investigator was a female clinician who had formal training and experience in conducting interviews for qualitative research as well as practical experience in physical activity programme implementation for this age cohort [32]. The approach to data collection involved asking set questions from a topic guide (S1 File) that also contained a set of prompts and alternative phrasing to promote reflection and meaningful answers as well as space for spontaneous discussion. Two versions of the topic guide, one for the programme attendees and another for community advocates and physical activity professionals, were designed by the research team based on principles of qualitative research and both were adapted after being initially piloted on a subset of the study population, some of whom subsequently took part in one to one interviews. The topic guide covered the central study questions and asked specifically about peer mentoring. Time and opportunity for general comments was provided in each instance. The investigator took field notes during each interview and focus group to describe context and non-verbal cues, and all were digitally recorded and transcribed verbatim.

Two methods of data collection were used in this study—one-to-one interviews and focus groups. The combined approach was a pragmatic decision as many of the professionals and advocates interviewed would have been unable to attend focus groups and were glad to have the opportunity to do a one to one interview, whereas the focus groups afforded the opportunity to those who had participated in physical activity as a group to reflect together on their individual and group experiences. While a one-to-one interview facilitates in depth understanding of an individual’s experience, a focus group may produce spontaneous interactions that can clarify, challenge, validate or criticise the context and components of a phenomenon [33]. A ‘side by side’ approach, whereby the analysis team constantly go over and back between the two types of datasets, comparing and contrasting responses can lead to a rich and more nuanced analysis, with one approach complimenting the other [34–36].

A total of 30 adults aged 50 years and over (77% female) attended four focus groups, including: a men’s activity group (n = 5); a women’s walking group (n = 6); a women’s cycling group (n = 9) and a group of physical activity leaders (n = 9). A further one-to-one interview was conducted with a female cycling mentor. Seven one-to-one interviews were conducted with physical activity professionals, six of whom were male. Ten one-to-one interviews were conducted with community advocates, of which eight were female, including a general practitioner, voluntary community leaders and state-employed managers.

The one-to-one interviews ranged from 34 to 59 minutes in duration and the focus groups lasted from 43 to 56 minutes. Three of the focus groups were all conducted in community centres, where the group met and, in some instances, continue to meet. Two of the groups were in areas considered to be ‘affluent’ and one in an area considered to be ‘disadvantaged’, according to the social deprivation index in the most recent census [37]. One-to-one interviews were conducted at the participants’ workplace, or, more commonly, over the phone.

### Data analysis

A team of seven researchers with experience in qualitative research (AOR, EGB, MC, SG, AC, LG, CW) from the disciplines of family medicine, psychology, physical activity, physiotherapy, nursing and social sciences, analysed the data following the principles of inductive thematic analysis. Initially two transcripts were picked at random; one from the focus groups and another from the one-to-one interviews and both transcripts were circulated to each member of the analysis team. Both transcripts were coded individually by every member of the team who subsequently met to clarify and agree on codes. In order to facilitate the comparison of coding between coders, the data were uploaded to NVIVO version 12 software. Two coders (AOR, MC) were appointed to code the next two transcripts, also picked at random. At the next meeting, these codes were reviewed, adapted and clarified. Subsequently, the two coders independently coded the remaining 18 transcripts, conferring after each transcript to ensure consistency and agreement and, when necessary, a third member of the team (EGB) met the coders to clarify uncertainties. This stepwise approach, involving all the team members early in the analysis, provided rigor to the process.

The entire team met for a third time, at which one coder (AOR) presented the codes to the others in order to familiarise the team with the organisation and categorisation of the data and to facilitate a shared understanding of its meaning. There was unanimous agreement that no new codes were emerging and that no further interviews were necessary. The meaning of each code was discussed at this meeting and the primary codes were grouped into higher-level codes. Two further meetings were held involving four members of the team to categorise the higher-level codes into themes. Themes were then compared for relatedness and grouped into overarching themes. The approach to data analysis was based on making meaning of the data so that it could be organised and categorised in order to conceptualise layers of understanding. Initial analysis was based on ‘rules of meaning’ whereby the researchers met and sought to agree understanding of what was expressed by participants. Higher level analysis was based on ‘unarticulated meaning’, the process of categorising data linked by themes that are understood by the researchers [38]. The spacing out of the process over time facilitated the researchers in reflecting on the data and interpreting its meaning in what has been compared to an incubation process [39].

## Findings

### Overview of findings

**Fig 1:**
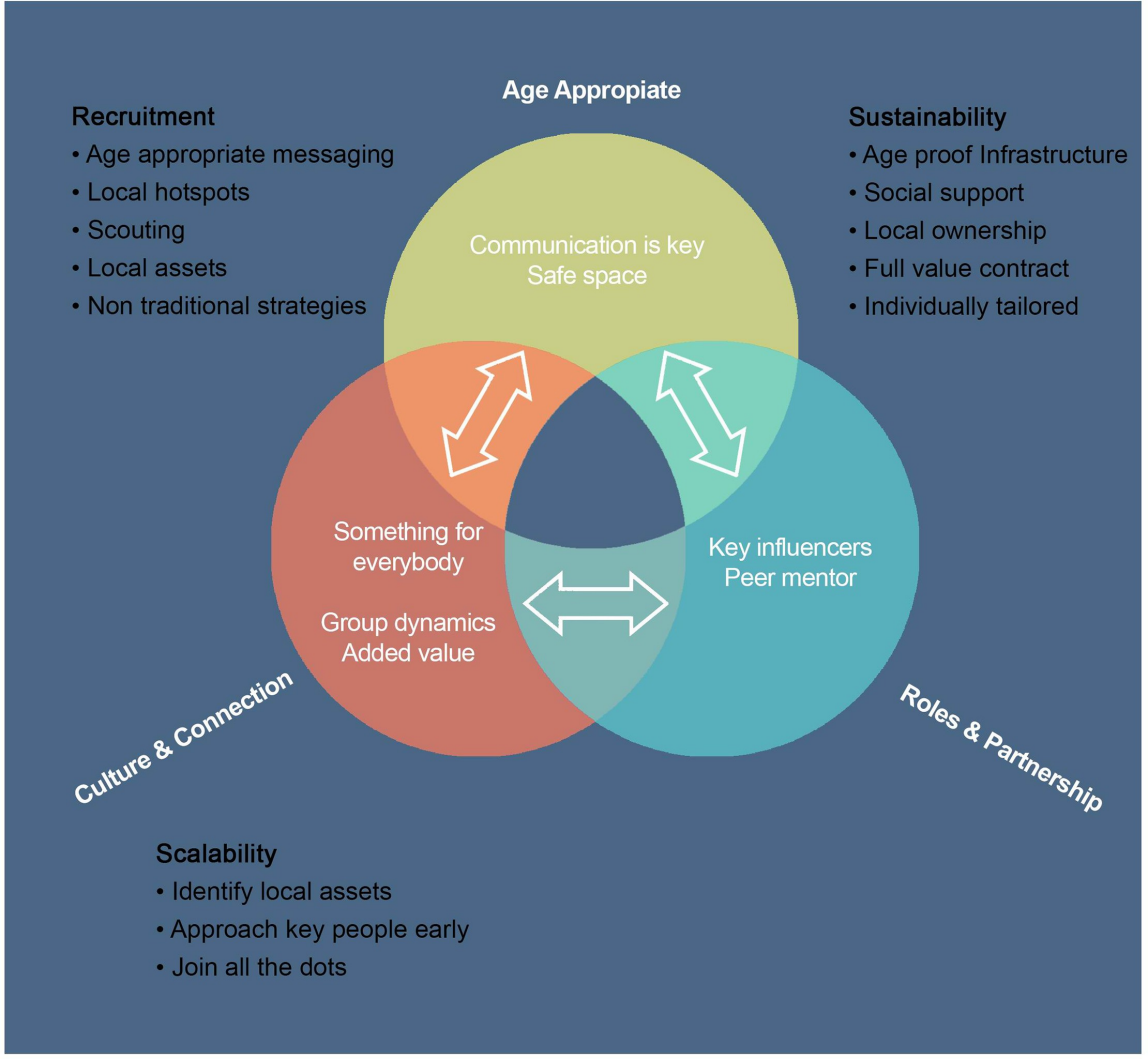
Relatedness of themes to each other and the research questions.

The data analysis produced three overarching themes, ‘age appropriate’, ‘culture and connection’ and ‘roles and partnerships’. Each had subthemes that often overlapped and were inter-related. The analysis below places the themes in the context of the objectives—recruitment, sustainability and scalability- and distils the essence of the meaning of each theme into a useable paradigm for designing future programmes. Fig 1 illustrates the relatedness of the themes and their relevance to the central research questions—recruitment, retention and scalability of interventions that promote physical activity.

### 1. Age-appropriate

This concept permeates the entire process of intervention planning, across recruitment, sustainability and scalability, ensuring that programmes are suitable for, and accessible to, the population. Its meaning is well illustrated by the term ‘age friendly’, which was used by a senior health promotion officer interviewed to describe the heightened awareness necessary when communicating with and creating the best environment for adults aged 50 years and older. Within it are two subthemes: ‘communication is key’ and ‘safe space’.

#### Communication is key

The consensus among all participants was that the manner in which this population is approached is ‘key’. Advocates for older adults and physical activity experts agreed that the content, style and attitude underlining the communication strategy must be appropriate in order to recruit and retain participants. Participants experienced in health promotion with adults aged 50 years and older advised suitable imagery, a reassuring tone and nomenclature that older people can identify with e.g. the use of a name like ‘fit sticks’ to describe a walking class, followed by a simple blurb describing the aim and content of the programme. A health promotion manager and also a trainer of tutors agreed, adding that the image of the fitness industry, that is perceived as being focussed on physical appearance is often seen as elitist and can be off-putting.

“Language is very important… [it] should be reassuring. Can they identify with the message?”

[physical activity professional, male]

“we must think about how to engage with older people and engage with them on a level playing field.”

[community advocate, female]

Multiple modes of communication were suggested, including online, social media, mass media and notices in local ‘hotspots’ where people aged over 50 years are likely to present e.g. the post office, church notice board, primary care centre. All identified word of mouth as particularly important. One programme attendee recounted having felt left out when she did not receive messages that were sent through an online platform. There was a sense among participants that traditional modes of communication, such as advertisements in newspapers and radio, online platforms and posters in gymnasiums, may exclude socially isolated (e.g. ethnic minorities) who would not have access to them, and to overcome this, planners must actively seek out and engage with such groups and individuals. Non-traditional methods of recruitment, including inter-generational approaches were suggested to inform harder to reach older adults, such as the very old, less educated and adults caring for family members full time in their own home. One instructor commented that success hinges on finding different channels in the locality:

“Recruitment from all angles… going to schools and asking children to talk to their grandparents”

[focus group—older men’s physical activity group]

“there’s a need for LSPs to go to the harder to reach groups and ask them what they want–travellers and ethnic minorities”

[community advocate, female]

#### Safe space

This subtheme explains the interplay between age, gender and the physical and social environment and how they relate to recruitment and sustainability. Study participants from all groups described age-specific considerations, such as poor acoustics, cold temperatures, unsteady chairs, poor building design and difficulty with transport.

A component of ‘safe space’ is ensuring that suitable activities are provided by a professional tutor with experience in working with this age cohort. One professional involved in programme design suggested that older tutors would be more suitable but this view was not held by most programme attendees who felt that age is irrelevant. The attitude of the instructor was the most highly valued facet by all in creating a positive environment for PA. All agreed that, for an instructor, empathy, checking in with attendees and responding to suggestions and needs were essential.

“they [instructors] must be authentic, sincere, passionate. Older people tend to be more discerning… they will know if the person is genuine about their well-being—they are very good ‘bulls**t detectors’.”

[community advocate, female]

Gender and space were a recurring dimension of this theme. Among the programme attendees, males found female-dominated classes off-putting, while a male-dominated class can be intimidating for women. In some instances, the sharing of information and bonding that took place beyond physical seemed to have been facilitated by gender segregation, e.g. issues such as comfortable underwear for cycling were discussed among participants in a women’s cycling programme.

“The (cycling) club was really male dominated… you were pushing yourself more than you were able… it was too aggressive. I fell and lost my confidence.”

[focus group—women’s cycling group]

### 2. Culture and connection

This theme explains the interplay of individual and social factors that influence sustainability of physical activity programmes. On one hand, enjoyment and camaraderie are drivers for participation, but on the other, reticence, fear and poor group dynamics can curtail them. It consists of three subthemes, which progress from the level of the individual to the group and beyond: ‘something for everybody’, ‘group dynamics’ and ‘added value’.

#### “Something for everybody”

Demonstrating that there is “something for everybody” is how one attendee of a walking group for older men emphasised the importance of inclusiveness. Many programme attendees expressed reluctance to engaging in present programmes or having declined to participate in the past. Self-image was a common barrier, with some programme attendees acknowledging a sense of fear and shame, holding a perception that they would not be able to keep up with other attendees, often based on previous negative experiences. Some programme attendees observed others dropping out of current programmes because the walking pace was too fast and for others illness and injury were barriers.

“A lot of people would have decided at a young age that they were no good at sport or that they didn’t like it and would have kept this perception for life”

[physical activity professional, female,]

In some instances, reaching a crisis point, such as all-time low fitness, loss of a partner or being overweight, acted as a motivator for action:

“I knew something had to be done”

[focus group–men’s physical activity group]

Recognising individual fears and personal contexts and creating a respectful and supportive atmosphere were valued by all. Most programme attendees identified the sense of community and camaraderie as the main factor in sustaining groups, but some considered the physical gains such as toning up, weight loss and better (physical) health as being primary. Beyond individual traits, community advocates identified those from poorer areas, with limited access to transport and those from ethnic minorities as being less likely to attend. Recognising individual fears and challenges and responding in a supportive way was seen as a lever for sustaining programmes.

“Sustainability is that slow change in the culture to change hearts and minds and to get people out doing exercise over time”

[community advocate, female,]

#### Group dynamics

Several community advocates suggested that key group members could promote positive group dynamics. One community advocate explained how a small number of enthusiastic and energetic people are needed to initiate and sustain a group. Programme attendees emphasised how they valued the availability of refreshments and breaks from exercise that facilitated interaction. Furthermore, physical activity professionals outlined strategies for incorporating opportunities for building relationships. Simple but important strategies were suggested by stakeholders from all three groups that would encourage good dynamics through fun and enjoyment: music, variety, games and keeping the tone light hearted.

“Taking a break in the middle and having tea and scones–social opportunities…the tutor [physical activity instructor] must facilitate that.”

[physical activity professional, male]

Other suggestions included introducing games into the activities to encourage networking, such as pairing up and discussing a suggested relevant topic. One focus group described how a buddy system emerged, whereby participants who had cars paired off with those that didn’t and drove them to the classes. This sense of solidarity was highly valued by most programme attendees:

“we are all like-minded”

[focus group—women’s walking group,]

Some differing opinions emerged regarding the formation of group identity. Most female programme attendees of cycling group supported the use of branding and unique merchandise to build strong identity and feel-good factor to the group. In contrast, a community advocate, who had herself taken part in a past programme, expressed reluctance about this, recognising it may lead to perceived exclusivity.

“one group bought themselves a uniform for cycling which is interesting… but that could turn some people off–some people do not like clubs and might feel that it is exclusive.”

[community advocate, female]

A more subtle facet of managing group dynamics that emerged was the role of competition. All the groups said that it existed to some degree, with most viewing it is a motivator. Notably, females favoured an internal competition with the self, whereas males tended to compete against each other. Women in particular, noted the concept of the more experienced attendees being able to reflect on their own journey and consequently show empathy for the less fit newcomers:

“*we know where they are coming from because we have been through it”*

[focus group–women’s cycling group]

#### “Added value”

The data repeatedly alluded to the added value beyond fitness gains. The story outlined by one LSP coordinator of a woman who reported that she was able to take the roast out of the oven again after participating in a physical activity programme illustrates how improved physical function and the confidence that the programmes instil bring about a powerful positive influence on daily living. It was as if the group dynamics described above carried into life outside of the programme, with one community advocate observing that “it can change their life around”. The ‘added value’ concept was understood in the context of social isolation, which was perceived by some interviewees as possibly the single biggest threat to older adults.

“I think the added value is huge. I didn’t have the energy to go but when I felt great. You feel that belonging. You feel that you would be missed if you didn’t go”

[community advocate, female]

“It’s not so much what I do; it’s the feeling I get from it”

[focus group–men’s physical activity group]

The value placed by this opportunity for connection that the programmes presented was evident in the active commitment to attend:

“Anything keep you from coming?”

“No.”

“Wild dogs wouldn’t keep him away!”

[focus group–men’s physical activity group]

A co-ordinator explained that trust and acceptance must be in place to create an atmosphere where participants get optimal value:

“the value contract is when everybody has a right of a voice, everybody has the right to say yes or no, everybody has the right to be challenged….”

[physical activity professional, male]

The other side of the ‘full value contract’ should emphasise both the importance of physical activity for the attendees as well as the potential that attendees themselves have to act as resources. Parity of esteem is illustrated in the following analogy:

“the people gave of their time and the hotel gave of their venue. Some participants from the older persons group gave back by teaching English to the staff.”

[community advocate, female]

Several of those interviewed from community advocate and physical activity professional groups indicated that those who most would benefit from this ‘added value’ were least likely to attend or to adhere to these programmes. Upscaling programmes to cater for people from less well-off areas, minority and more socially vulnerable individuals, must overcome a range of challenges, from transport to language barriers.

### 3. Roles and partnerships

This overarching theme explains the process of how key collaborations may be identified and managed in order to optimise the three distinct concepts of recruitment, sustainability and scalability. It consists of two subthemes: ‘key influencers’ and peer mentors.

#### Key influencers

Interviewees from the physical activity professional and community advocate groups perceived ‘key influencers’ as individuals from within the community who could act as either a trusted source of information or as a role model, e.g. local doctors, shopkeepers, priests and school teachers. Public health nurses, were also considered key influencers for harder to reach groups such as older adults who care for family members in their homes. Married couples joining together were viewed as key influencers and a means of breaking down traditional trends of males not joining groups dominated by women.

“I remember one day the local GP came in—he got down and did the exercises with them. They were chuffed and honoured. They know how busy he is and it showed that he cared that he would give up his time.”

[community advocate, female]

Programme attendees tended to view ‘key influencers’ in a subtler way. Female attendees often joined with a friend, whereas males sometimes joined a programme as part of an existing group. In that way, men were often attending physical activity programmes as an extension of something else. The drawback is that those that are less likely to have friends or to be in groups may be less likely to join. The term “scouting”, described by one physical activity professional, illustrates how often there is an initial reticence to joining and many will wait and see how a friend gets on before joining themselves. In this way, attendees become recruiters themselves and some reported that as programmes grew in size, offshoots developed.

“I wasn’t going to come on my own–we do most things together. this is kind of auxiliary to what we do anyway”

[focus group–men’s physical activity group]

Local organisations can be ‘key influencers’ and sporting organisations, the Gaelic Athletic Association in particular, were viewed as important community-based organisations that could share resources but could also act as a conduit for reaching out to inactive people. Some interviewees with experience in planning physical activity programmes, stressed the importance of identifying local infrastructure and involving stakeholders early in the process. Others recognised that, in many regions, substantial initiatives and effective groups are ongoing but that upscaling to a national level would require high level and local co-ordination.

“We’ve got all these wonderful dots around the country but we’ve got to start joining up these dots.”

[community advocate, female]

#### Peer mentor

Interviewees, from all stakeholder groups, in response to direct questioning about peer mentoring, were initially uncertain about the meaning but, after discussion, could appreciate its value. Programme attendees recounted how important a role model or mentor was to them in their early stages:

“Hearing the testimonies of others is what kept me going”

[focus group- women’s cycling group]

A range of roles for a peer mentor was proposed, including organisational and as a subtle mediator between the group and the tutor. It was felt that what was most important was striking the balance between an individual who has presence and enthusiasm but does not dominate. In the context of appointing a peer mentor from a group, some were concerned about burnout, volunteer saturation and petty jealousies. Such pitfalls could be minimised by rotating roles and providing incentives for peer mentors. The concept of communities of practice emerged as both an incentive and support for peer mentors.

“definitely some education and support… to develop a community of practice that the peer mentors have an opportunity to come together and share information”.

[community advocate, female]

Physical activity professionals identified a potentially significant role for peer mentors in upscaling programmes, based on their experience with programmes that had failed due to lack of “local ownership”. Successful programmes tended to have resulted from requests made by strong drivers from within the community. It would seem that programme organisers, in this case the LSPs, preferred a pro-active first point of contact who will open doors to the community. The peer mentor concept was generally viewed as the lever to achieving local ownership–the person or persons who would entice, organise and encourage others in the community to participate and continue with the programme and thereby securing both programme sustainability and scalability.

“Often programmes will fall flat if there is no local ownership… give ownership to groups and make them sustainable by self-funding”

[physical activity professional, male]

## Discussion

### Summary of findings

Valuing people and building relationships were principles common to all themes. The key messages for successful recruitment, sustainability and scalability were understanding that the target population has unique needs that must be catered for when planning interventions, communicating messages and choosing personnel. Social connection and group cohesion permeate through the themes.

### Comparison to the literature

The importance of the social element to physical activity emerged strongly from the data, including the reticence and personal fears that many adults have and how they are often barriers to participation. Fear and stigma are previously reported barriers [40] and social support has been suggested as a solution to overcome them by research with professional physical activity instructors working with older adults, in particular [41]. The consensus among participants in this study was that creating a safe environment can enable adults to overcome barriers; a concept that is also very important for children [42]. To have a ‘safe space’ is a lifelong determinant of recruitment to and sustainability of physical activity programmes, it seems. Similarly, the analysis demonstrates that poor group dynamics can negatively affect programme sustainability, with most agreeing that the instructor’s primary role was to foster this positive social environment. Our analysis introduced the term ‘scouting’, the ongoing recruitment as the physical activity programme unfolds, of new attendees by those who had joined initially. This phenomenon, whereby more active participants are encouraging more reticent observers, is underpinned by the concept of social support from the literature, whereby people who are supported are more likely to undertake physical activity [43]. Its importance relates to recruitment and scalability of physical activity programmes.

Individual factors such as illness, isolation, very old age, gender as well as belonging to social minority groups were identified as barriers to recruitment and sustainability and are consistent with barriers reported in the literature [44]. Study participants described how some people are not reached by traditional recruitment methods and highlighted techniques to overcome this. The concept of couples joining together to encourage others has been reported and is often a complex dynamic, as spouses tend to have different preferences based on their gender [45]. Other approaches, including the local GP attending a class with his patients and the public health nurse recruiting isolated adults who care for a spouse or family member in their own home, are novel and contribute to the literature on recruitment and scalability.

Boredom and poor motivation were commonly encountered by all groups, who suggested that varying the content and difficulty of the physical activity helped to retain their interest. Furthermore, making it light and fun and emphasising the ‘added value’ that extended beyond the class time were recommended levers to overcome these. These findings are consistent with previous research on programme sustainability recommending enjoyable group-based activities that promote self-efficacy [46]. They are also consistent with the findings of recent research that encourage a shift in focus to life satisfaction and sense of purpose for older adults [47].

In addition, professionals noted the importance of tailoring physical activity to the level of the individual. This is important in the context of adults aged 50 years and over being a diverse group affected by gender and social roles [48] and the importance of involving programme attendees in the planning and conducting of public health interventiosn to ensure buy-in and commitment [49]. Physical gains may be the principal motivator for joining walking groups but social connectedness is the main factor in sustaining them [50]. Study participants appreciated that PAP facilitators checked in with them, that they appeared to be concerned about their capability and that they were willing to respond. Participant-centeredness is particularly important in the training of facilitators in the context of PAP sustainability. Our findings align with research highlighting the role of leadership, promoting group cohesion and individualised interventions based on behavioural theories [51]. Furthermore, a systematic review of reviews concluded that these programmes should accommodate social and individual needs of adults aged 50 years and over who attend PAPs [52].

Interestingly, a single participant from the physical activity professionals group believed that younger facilitators would not be acceptable to older adults, while the attendees themselves were clear that age is irrelevant. Another source of disagreement emanated from within the attendee group with females expressing negative sentiments regarding competition but males perceiving competition as a motivator. On the subject of gender, the physical activity professionals and community advocates did not see it as a significant factor, whereas both male and female attendees expressed a preference for gender segregation, but for different reasons.

A key factor identified for scalability and sustainability of physical activity programmes was local ownership and most agreed that success hinged upon the presence of key people in the locality encouraging and supporting others. This concept of partnerships with organisations beyond the health sector is consistent with published research on scalability [1]. In this context, the term ‘peer mentor’ was introduced by the interviewer, and, while there were different opinions as to what extent of the role should be, most agreed that the concept was important. Peer mentors can help to promote and maintain participation in physical activity programmes [53]. Based on our analysis, an intervention with a peer mentoring component should clearly delineate the role of the peer mentor and the support system before recruitment begins. Study participants acknowledged that while every community will have different strengths, it is necessary to approach peer mentors and other community assets very early and involve them in the planning in order to successfully recruit for and subsequently upscale programmes. Adapting to the local context is essential to promote physical activity interventions in socially disadvantaged areas [54].

### Strengths and limitations

A multi-disciplinary team of experienced qualitative researchers met on five separate occasions to discuss, argue and agree on the meaning of the data, adding substantial rigour to the analysis. The study involved the full spectrum of professionals involved in physical activity programmes, community advocates and a diverse group of PAP attendees who offered a wide range of experiences of the subject. The study was limited to the Irish setting and the programme attendees came from communities with differing social deprivation indices, indicating that a range of socio-economic groups were represented. Individual demographic data, including measures of age, socio-economic group, disability and ethnicity were not recorded for each participant. As recruitment was via email and word of mouth, it is possible that a significant number of adults aged 50 years and over did not participate due to digital exclusion. Finally, this study does not describe the perspectives of adults aged 50 years and older who have never attended such programmes, but study participants were asked for their views on strategies that would help recruit inactive adults.

### Implications for future research and practice

Future research into how behavioural change techniques, such as cognitive-behavioural skills, social support and group dynamics, can influence sustainability of physical activity programmes is warranted. Research should investigate how state departments, including health, sport and transport can collaborate to facilitate upscaling of such interventions so that they become embedded into the system. In clinical practice, clinicians, public health nurses and all health workers in contact with adults aged 50 years and older must become ‘key influencers’ by taking a proactive approach to physical activity promotion. Public health strategists planning to recruit from this age group should use age appropriate messaging, invite local ‘assets’ to act as champions and consider techniques like scouting. Those involved in designing physical activity programmes should ensure that professionals understand concepts such as the full value contract, local ownership and individual tailoring and that infrastructure is age appropriate. Anyone planning to upscale projects might consider identifying local assets and involving key participants at the earliest phases of development, in particular those identified as key stakeholders and potential peer mentors.

## Conclusion

This study has deepened understanding of how all stakeholders experience community-based physical activity programmes. The findings of this study have informed the development of the Move for Life intervention which has been used as part of a feasibility and pilot trial recruiting over 700 adults aged 50 years and older in the West of Ireland. The findings of this qualitative study can be used to inform strategies for recruitment, sustainability and scalability of further complex interventions to promote physical activity.

## Supporting information

S1 Table Consolidated criteria for reporting qualitative research (COREQ): 32 item checklist. (PDF)

S1 File Interview guide. (DOCX)
