# Supplementary material for: How to improve recruitment, sustainability and scalability in physical activity programmes for adults aged 50 years and older: A qualitative study of key stakeholder perspectives
Source: PLoS One. 2020 Oct 29;15(10):e0240974. doi: 10.1371/journal.pone.0240974 (PMC7595437; doi:10.1371/journal.pone.0240974)
Supplement: S1 File — (DOCX) [file pone.0240974.s002.docx]

**Supplementary file 1**

**Move for Life - Participants’ Topic Guide Questions**

*Seeking information on Recruitment, Retention, Peer Mentors and Sustainability.*

Q1: Can you tell me a little bit about your experience with (activity) here in (venue)?

Q2: Have you been able to attend the majority of the sessions?

What makes you keep coming? Is there anything that might stop you from continuing?

Will you keep going? Why? Why do you think people attend?

Q3: You have been coming here for a while now. How does this feel to be part of something like this?

Q4: Some people say that the exercise programmes work better when the mentor comes from the same group. What do you think?

Q5: What factors do you think could be improved in order to increase the uptake of the programme here?

Q6: Think back to a time when someone asked you to do a physical activity. Why did you go or not go?

Q7: What advice would you give us for people who are inactive over 55?

How would we reach them? How to motivate people to participate?

Q8: These are examples of how we advertise and recruit people into our programme. What do you think? What are we missing?

Q9: Final question: In an ideal world is there anything else you would like to add?

Is there anything else you would like to tell us about?

**Move for Life - Stakeholders’ Topic Guide Questions**

*The focus is: Recruitment, Retention and Scalability/Sustainability.*

*Part 1: Understanding participation in four existing physical activity programmes delivered by the Sports Partnership in the Hubs in Clare and Limerick*

- *Men on the Move*
- *Women on Wheels (Limerick) / Bike for Life (Clare)*
- *Go for Life / Older adults exercise classes*
- *Get Ireland Walking*

Q1: Can you tell me briefly about your role and how long have you been working in this role?

Does your work involve promoting or supporting physical activity for the 50+?

Q2: Based on your experience (of these 4 programmes) can you give me 2 or 3 factors that are critical for the success of these types of groups?

How do you gauge the success of these groups?

What are some factors that would help retention of the participants?

Q3: What are the supports and infrastructure required in order to put a successful programme in place? And / or referral pathways?

Q4: What community factors or key people can energise and support these types of programmes?

Q5: What would help these programmes to run in more places around the country?

Q6: Are there any groups that are benefitting more than others and why?

How could we improve the reach to the disadvantaged?

*PART 2: Looking at the new Move for Life peer mentor intervention or programme which will be described before the interview.*

Q7: Would you use peer mentor or another term to describe a person from within a physical activity group that takes on an additional helping role?

Q8: How would you see the role of the peer mentor in the 4 programmes?

What will they do?

How do you find the peer mentor and what are the qualities needed?

What support would a peer mentor require?

How could we offer supports in a more structured way to a peer mentor to help sustain the programmes in the long term?

Q9: What changes would be needed so this Move for life peer mentor intervention could be introduced in more places around the country?

What would allow the Move for Life intervention to sustain itself or keep going?

For this intervention to run nationally and grow?

Q10: Are there any barriers you see to running the Move for Life intervention and how do we overcome those?

Q11: In an ideal world what would you do to recruit/get in the over 50s and keep them going to the physical activity programmes?

What would you need to signpost /refer older people to these programmes?

What would you hope this new peer mentor intervention would achieve for over 50s?

Q12 Final question: Is there anything else you would like to add?
